# A comparison of the effect of procedural pain on cerebral oxygen saturation between late preterm and term infants

**DOI:** 10.1038/s41372-024-01978-4

**Published:** 2024-05-24

**Authors:** Ittichote Amornjiraporn, Supranee Rugsapol, Peerawit Thanasarnpaiboon, Bosco Paes, Ratchada Kitsommart

**Affiliations:** 1 Department of Pediatrics, Faculty of Medicine Siriraj Hospital, Mahidol University, Bangkok, Thailand; 2 Nursing Division, Department of Pediatrics, Faculty of Medicine Siriraj Hospital, Mahidol University, Bangkok, Thailand; 3 Division of Neonatology, Department of Pediatrics, McMaster University, Hamilton, ON Canada

**Keywords:** Paediatrics, Neurological manifestations

## Abstract

**Objectives:**

We prospectively compared cerebral oxygen saturation (CrSO2) and pain score changes during procedures in late preterm (LPT) versus term infants.

**Methods:**

Near-infrared spectroscopy, pulse oximetry, Neonatal Infant Pain Scale (NIPS) and Premature Infant Pain Profile-Revised (PIPP-R) scores were assessed and CrSO2 data analyzed.

**Results:**

Thirty infants in each group were evaluated. LPT infants displayed a milder significant drop in Minimum post-procedural CrSO2 and smaller Maximum-Minimum post-procedural CrSO2 disparity. CrSO2 minute changes between the groups were non-significant. Moderate correlations were observed in both groups between NIPS and Minimum post-procedural CrSO2, and a moderate correlation was found in the Maximum-Minimum post-procedural CrSO2 difference in LPT infants. No correlation between PIPP-R and CrSO2 values was noted.

**Conclusion:**

LPT and term infants demonstrated decreased CrSO2 in response to painful procedures. Correlations between CrSO2 and PIPP-R or NIPS scores were poor to moderate, reflecting the complex nature of these associations relative to gestational age.

## Introduction

Pain is a response to tissue injury or events that cause tissue damage, to protect the organism from further harm [1]. It relies on various components of the nervous system, including nociceptors, sensory nerve fibers, the spinal cord, and the brain [2]. Consequently, individual infants may perceive pain differently, even when subjected to similar proximal stimuli. Significant associations exist between the number of painful procedures and markers of white matter diffusivity and brain metabolites in both white matter and subcortical gray matter of preterm infants at term corrected age [3]. Additionally, a significant correlation prevails between the number of painful procedures performed during the course of neonatal intensive care and reduced intelligence and brain development in childhood [4]. Painful procedures in extremely preterm infants not only alter the perception of pain but also contribute to an increased sensitivity to pain and may be linked to the development of chronic pain symptoms at an older age [5]. Therefore, it is imperative to avoid or minimize painful procedures, as well as assess the level of pain to provide appropriate care or administer suitable pain-reducing medications. The investigation of the impact of procedural pain has predominantly focused on very preterm infants [6–8].

Late preterm (LPT) infants, defined as those born between 34 and 36 weeks of gestation, represent another vulnerable subgroup who are exposed to diverse painful procedures soon after birth [9]. Due to intense synaptogenesis and dendritic arborization that occurs in the late preterm period, these infants are characterized by extensive connectivity linked to crucial brain functions [10]. Moreover, evidence suggests that LPT infants have a higher propensity for long-term brain-related complications and developmental challenges compared to full-term infants [11, 12]. Although LPT infants are of modest body weight and possess physical characteristics resembling those of their full-term counterparts, they are more likely to undergo procedures such as venipuncture and heel stick blood sampling, and administration of intravenous or intramuscular medications. Consequently, there has been a tendency to overlook the assessment and treatment of pain in this cohort.

Given the limitation that infants cannot directly communicate their pain, several assessment tools have been devised, namely, the neonatal infant pain scale (NIPS) and the premature infant pain profile-revised (PIPP-R) scores, to evaluate pain that incorporate facial expressions along with other physiological parameters. Researchers have identified an association between cerebral oxygen saturation (CrSO2) and neonatal pain perception [6, 13–15]. Due to the period of brain immaturity in LPT infants, we anticipated that changes in CrSO2 in response to painful stimuli may be greater in LPT infants compared to their term counterparts. This is because of the increased oxygen consumption and less mature hemodynamic coupling in LPT infants compared to term infants. Therefore, our primary objective was to compare the change in CrSO2 during painful medical procedures between LPT and term infants. The secondary objective was to explore the correlation between the change in CrSO2 and clinical pain scores (PIPP-R and NIPS).

## Methods

A prospective cohort study was conducted in the high-risk nursery of Siriraj Hospital, a tertiary-care facility in Thailand. Consistent with our institutional protocol, hemodynamically stable preterm infants <37 weeks gestational age (GA), not requiring positive-pressure ventilation for respiratory compromise, are admitted to the high-risk nursery. Very-low birthweight stable infants (birthweight <1500 g) are allocated to intermediate care, while those manifesting respiratory compromise or hemodynamic instability are admitted to the neonatal intensive care unit. Determination of GA is established through maternal ultrasonographic dating during the first trimester, with postnatal clinical examination serving as an alternative in cases where ultrasonographic data are unavailable.

Infants included in the study encompassed the following criteria: (1) inborn within 34 to 42 weeks GA and birthweight ≥ 1500 g, (2) postnatal age <14 days, (3) Stable respiratory status without positive-pressure ventilation, (4) hemodynamically stable without use of vasoactive agents. Exclusion criteria comprised infants exhibiting signs of neonatal encephalopathy such as abnormal movements, hypotonia, or apnea; those with severe congenital anomalies; and individuals for whom placement of a near-infrared spectroscopy (NIRS) sensor was unfeasible. To uphold biological diversity, each infant was singularly enrolled. Additionally, in multiple pregnancies, only one twin was enrolled to mitigate potential bias in genetic factors. The LPT and term groups comprised infants of 34–36 weeks and 37–42 weeks GA respectively.

The care of neonates and the decisions regarding the modality of blood sampling, either through heelstick or venipuncture, resided with the clinical discretion of the attending physician. A single INVOS™ 5100 C device was utilized to measure CrSO2 in conjunction with the OxyAlert™ sensor. The infant was placed supine on a radiant warmer for the procedure, a sensor was applied to the frontoparietal area, and was wrapped with Easifix™ cohesive bandage around the head to ensure fixation. Oxygen saturation (SpO2) and heart rate were concurrently measured using a Nellcor™ Bedside SpO2 Patient Monitoring System and MAXN sensor, with signals affixed to the right hand. The procedure was executed using a standardized protocol, incorporating simultaneous video documentation for subsequent pain evaluation. Continuous CrSO2 monitoring was conducted for a period of 10 min before the initiation of the procedure and continued for at least 10 min post-procedure. The accepted reference range for CrSO2 values was 60 to 80%.

The CrSO2 data of every 20-second average retrieved from the equipment were documented and stored in the data collection form for subsequent analysis. Data points beyond three times the interquartile range (IQR) were excluded as outliers. Recording a video to distinctly capture facial expressions was imperative for the comprehensive assessment of pain scores, in conjunction with heart rate and SpO2 values. Pain assessments were conducted through the analysis of video recordings employing the PIPP-R score [16] and NIPS [17]. These evaluations were carried out by a sole assessor (SR), who maintained a degree of independence by not being directly involved in patient care. This ensured both the reliability and impartiality of the assessment process.

### Statistical analysis

Due to the absence of established reference values regarding expected variations in CrSO2 during painful procedures in LPT and term infants, a pragmatic approach was employed to guide the selection of 30 infants for each group as the sample size. This predetermined sample size ensured a robust statistical power of 99%, allowing for the examination of the correlation coefficient *(r*) between clinical pain scores and the change in CrSO2 at a significance level of 0.8. Moreover, it maintained a power exceeding 80% even if the value of *r* equals 0.6, which falls well within the range of *r* values reported in the recent study by Kumar et al. [6]. We estimated that the sample size would adequately cover a range of moderate to higher correlation coefficients.

Infant demographic characteristics are reported in number (percentage), mean ± standard deviation (SD), or median [25th percentile, 75th percentile; P25, P75] where appropriate. The onset of the procedural intervention was defined as the point at which puncture was initiated or catheter insertion through the neonatal skin began. The computation of each CrSO2 variable was executed using scientific computation libraries in the Python programming language, specifically SciPy version 1.11.3, within the Jupyter Notebook environment. CrSO2 variables were defined as follows:

1. *Baseline CrSO2*: Median and interquartile range (IQR) of CrSO2 from the 10-min period preceding the commencement of the procedure.
2. *Post-procedural CrSO2 (PoP CrSO2)*: Median and IQR of CrSO2 from the 10-min period following the commencement of the procedure.
3. *Maximum post-procedural CrSO2 (Max PoP CrSO2)*: The maximum CrSO2 values during the 10-min period following the commencement of the procedure.
4. *Minimum post-procedural CrSO2 (Min PoP CrSO2)*: The minimum CrSO2 values during the 10-min period following the commencement of the procedure.

Statistical analyses were performed using IBM SPSS Statistics 29.0.1.0 software, with a predefined significance threshold set at *p* < 0.05. Demographic characteristics between LPT and term infants were compared using chi-square, Fisher exact, independent *t*, or Mann-Whitney U tests. Changes in CrSO2% values from baseline CrSO2 to PoP CrSO2 are presented as mean ± SD and compared between groups using the independent *t*-test or Mann-Whitney U test. The variation in PoP CrSO2 at each minute within the LPT or term infant groups, relative to baseline CrSO2, was examined using the Friedman chi-square test based on non-normal distribution.

The initial 20 patients were utilized to evaluate the reliability of the PIPP-R scores and NIPS by the single assessor. This assessment was conducted using the intraclass correlation coefficient (ICC) and a 95% confidence interval (95% CI), employing a single-rating, absolute agreement, 2-way mixed effects model. Additionally, the correlation between PIPP-R scores (ranging from 0 to 18) or NIPS scores (ranging from 0 to 7) and changes in CrSO2 was examined using the Spearman correlation coefficient (*r*).

## Results

Between August 1st 2022 and November 30th 2023, the study enrolled 30 LPT and 30 term infants. Demographic characteristics are detailed in Table 1. Apart from a lower GA, LPT infants exhibited a significantly higher rate of antenatal dexamethasone administration (76.7% vs. 3.3%, *p* < 0.001) and a lower mean birth weight (2432.1 ± 392.6 vs. 2967.7 ± 482.0, *p* < 0.001). The median postnatal age in the LPT group was earlier than the term group (4.1 [1.0, 6.5] vs. 11.5 [3.0, 55.0] hours, *p* = 0.02). Venipuncture was the most common procedure in both groups (50% and 53.3% in LPT and term infants, respectively), with no significant difference in procedural types between the groups. Administration of oral sucrose solution occurred in 2 infants (6.7%) of in the LPT group and 3 infants (10%) in the term group, with no statistically significant difference observed (*p* = 1.00).

**Table 1:** Maternal and infant demographic characteristics.

Characteristics	Late preterm (*n* = 30)	Term (*n* = 30)	*p*
**Mothers (*n*** = **60)**			
Age (year)	30.9 ± 6.2	31.1 ± 5.4	0.90
Hypertension-related disorders	3 (10.0%)	0 (0%)	0.24
Diabetes	6 (20.0%)	12 (40.0%)	0.09
Intrapartum systemic analgesia	5 (16.7%)	8 (26.7%)	0.35
Magnesium sulfate administration	2 (6.7%)	1 (3.3%)	1.00
Antenatal dexamethasone	23 (76.7%)	1 (3.3%)	<0.001*
Cesarean section	18 (60.0%)	13 (43.3%)	0.20
**Infants (*n*** = **60)**			
Gestational age (week)	35 [35,36]	38 [37,39]	<0.001*
Postnatal age (hour)	4 [1.0,6.5]	11.5 [3.0,55.0]	0.02*
Birth weight (*g*)	2432.1 ± 392.6	2967.7 ± 482.0	<0.001*
Male sex	14 (46.7%)	21 (70.0%)	0.07
5-min Apgar score	9.5 [9,10]	10 [9,10]	0.66
Small-for-gestational age	2 (6.7%)	7 (23.3%)	0.15
Large-for-gestational age	1 (3.3%)	3 (10.0%)	0.61
**Types of procedure**			0.06
Heel stick	10 (33.3%)	3 (10%)	
Intramuscular injection	5 (16.7%)	10 (33.3%)	
Orogastric tube insertion	0 (0%)	1 (3.3%)	
Venipuncture	15 (50.0%)	16 (53.3%)	

Data are presented as mean ± standard deviation, number (%), or median [25th percentile, 75th percentile]. *p < 0.05 is statistically significant.

No outliers as per definition were identified in the CrSO2 analysis. Table 2 delineates the CrSO2 values and alterations in response to a painful procedure. Baseline CrSO2 values in the LPT and term groups were 80.0 ± 5.3% and 81.5 ± 6.4%, respectively which was statistically non-significant (*p* = 0.33). Despite both LPT and term groups showing a minimal decrease in the 10-min averaged PoP CrSO2 (−0.5 ± 3.7% vs. −0.8 ± 4.2%, *p* = 0.71), infants in the LPT group exhibited a significantly lesser drop in Min PoP CrSO2 from baseline CrSO2 (−5.0 [−8.6, −3]% vs. −8.5 [−14.9, −4]%; *p* = 0.02) and a smaller disparity between Max and Min PoP CrSO2 (9 [6,13]% vs. 15.5 [9,18.3]%, *p* = 0.001) compared to the term group. Additionally, the LPT group demonstrated a significantly smaller IQR in PoP CrSO2 than the term group (3.8 [2.8,4.9] % and 4.7 [3,8.5] %, *p* = 0.02). Two LPT infants and 3 term infants had PoP CrSO2 levels below 60%, but no statistically significant difference was observed (*p* = 1.00). Additionally, neither infant exhibited PoP CrSO2 levels below 55%. LPT infants exhibited a non-significant tendency towards a slower time to Min PoP CrSO2 compared to the term group (3.8 [1.5, 6.0] vs. 3.1 [1.7, 6.5] minutes, *p* = 0.98).

**Table 2:** Comparisons of cerebral oxygen saturation during the procedure between late preterm and term infants.

	Late preterm (*n* = 30)	Term (*n* = 30)	*p*
Baseline CrSO2 (%)	80.0 ± 5.3	81.5 ± 6.4	0.33
IQR of baseline CrSO2 (%)	2.8 [2,4.4]	4 [2,4.8]	0.06
Average 10-min of PoP CrSO2 (%)	79.5 ± 6.2	80.6 ± 6.8	0.51
IQR of PoP CrSO2 (%)	3.8 [2.8, 4.9]	4.7 [3, 8.5]	0.02*
Max PoP CrSO2 (%)	84.5 [79.8, 89]	87.5 [80,91]	0.17
Min PoP CrSO2 (%)	74.0 ± 7.6	71.5 ± 7.3	0.20
PoP CrSO2 < 60% (%)	2 (6.7%)	3 (10%)	1.00
PoP CrSO2 - baseline CrSO2 (%)	−0.5 ± 3.7	−0.8 ± 4.2	0.71
Max PoP CrSO2 - baseline CrSO2 (%)	4.0 ± 3.5	4.7 ± 5.4	0.55
Min PoP CrSO2 - baseline CrSO2 (%)	−5.0 [−8.6, −3]	−8.5 [−14.9, −4]	0.02*
Max PoP CrSO2- Min PoP CrSO2 (%)	9 [6,13]	15.5 [9, 18.3]	0.001*
Time to Max PoP CrSO2 (minute)	4.8 ± 2.6	5.1 ± 2.4	0.64
Time to Min PoP CrSO2 (minute)	3.8 [1.5, 6.0]	3.1 [1.7,6.5]	0.98

*CrSO2* cerebral oxygen saturation, *IQR* interquartile range, *baseline CrSO2* 10-min average of median CrSO2 preceding the initiation of the procedure, *PoP CrSO2* 10-min average of median CrSO2 during the 10-min post-procedure, *Max PoP CrSO2* maximum CrSO2 during the 10-min post-procedure, Min PoP CrSO2; minimum CrSO2 during the 10-min post-procedure.

Data are presented as mean ± standard deviation, number (%), or median [25th percentile, 75th percentile]. *p < 0.05 is statistically significant.

**Fig. 1:**
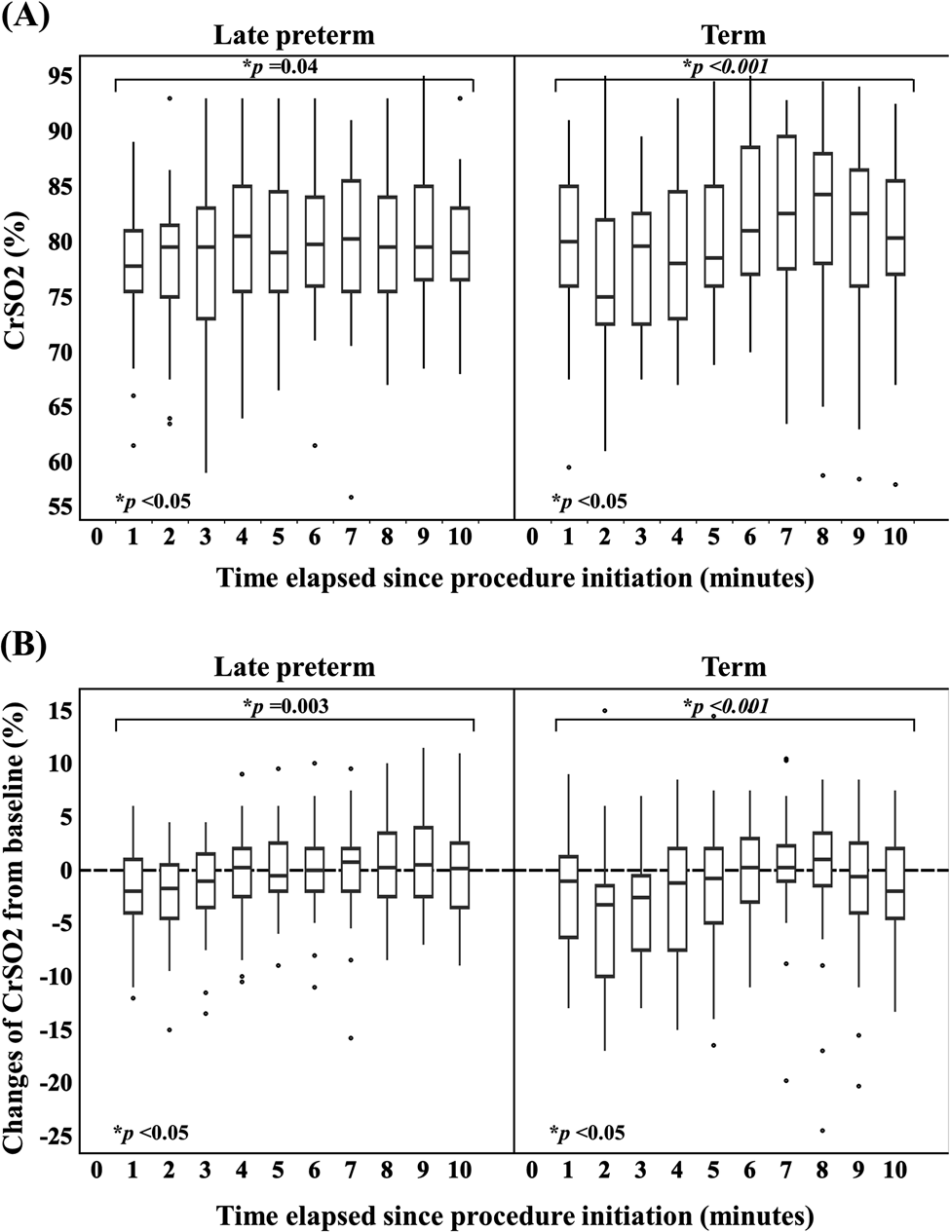
Comparison of cerebral oxygen saturation (CrSO2) changes at each minute interval during the procedure among late preterm and term infants. **A** presents the comparison of CrSO2, and **B** depicts the comparison of changes in CrSO2 from baseline. (*p-value < 0.05 is statistical significance). CrSO2 cerebral oxygen saturation.

The PoP CrSO2 showed statistically significant differences within both the LPT and term groups (*p* = 0.04 and < 0.001, respectively). Figure 1A depicts the PoP CrSO2 at minute intervals from minute 1 to minute 10, revealing that the term group initially experienced a decrease in CrSO2 followed by a gradual increase, a pattern not observed in the LPT group. However, when comparing PoP CrSO2 for each minute between the LPT and term groups, no statistically significant differences were noted at any post-procedure time (Supplemental Table 1; *p* > 0.05 for all). Figure 1B illustrates the alteration of PoP CrSO2 from baseline at each minute interval during the procedure between the groups. The magnitude of changes was significantly different within both the LPT and term infant groups (*p* = 0.003 and < 0.001, respectively). Nevertheless, comparisons of the degree of changes at each minute interval between the groups were not statistically significant (Supplemental Table 2; *p* > 0.05 for all).

The intraclass correlation coefficients for NIPS and PIPP-R scores were 0.81 (95% CI: 0.57, 0.92; *p* < 0.001) and 1.00, respectively. These findings demonstrate good intra-rater reliability of the assessor in the evaluation of pain scores. Median PIPP-R scores were 10.25 [4.4,13.6] and 11.75 [7,15] in the LPT and term groups which was non-significant (*p* = 0.25). Median NIPS scores were also similar between the groups (6.5 [3,7] vs. 7 [3.5, 7], *p* = 0.34). Table 3 outlines a comprehensive analysis of the intricate relationship between clinical pain scores and CrSO2. Within both the LPT and term group procedures, no discernible associations were observed between PIPP-R or NIPS scores and the 10-min averaged PoP CrSO2 and Max PoP CrSO2. Notable findings emerged in the term group, where a significant moderate negative correlation was established between NIPS scores and the reduction in PoP CrSO2 from baseline CrSO2 (*r* = −0.43, *p* = 0.02), as well as Min PoP CrSO2 (*r* = −0.39, *p* = 0.03), and the decrease in Min PoP CrSO2 from baseline CrSO2 (*r* = −0.41, *p* = 0.03). Conversely, the LPT group exhibited a moderate negative correlation between NIPS scores and Min PoP CrSO2 (*r* = −0.37, *p* = 0.04), coupled with a moderate positive correlation in the difference between Max PoP CrSO2 and Min PoP CrSO2 (*r* = 0.55, *p* = 0.002). Importantly, a lack of correlation was noted between PIPP-R scores and CrSO2, except for a moderate positive correlation with the disparity between Max PoP CrSO2 and Min PoP CrSO2 (*r* = 0.48, *p* = 0.01).

**Table 3:** Spearman correlation coefficients between the pain scores and cerebral oxygen saturation.

CrSO2 variables	Late preterm (*n* = 30)				Term (*n* = 30)			
	PIPP-R	*p*	NIPS	*p*	PIPP-R	*p*	NIPS	*p*
Average 10-min of PoP CrSO2 (%)	−0.13	0.48	−0.19	0.31	−0.13	0.49	−0.28	0.14
PoP CrSO2 - baseline CrSO2 (%)	0.07	0.70	0.00	0.98	−0.33	0.08	−0.43	0.02*
Max PoP CrSO2 (%)	−0.02	0.91	−0.02	0.93	−0.02	0.91	−0.24	0.21
Min PoP CrSO2 (%)	−0.29	0.13	−0.37	0.04*	−0.23	0.22	−0.39	0.03*
Max PoP CrSO2 - baseline CrSO2 (%)	0.31	0.09	0.32	0.08	−0.16	0.40	−0.32	0.09
Min PoP CrSO2 - baseline CrSO2 (%)	−0.26	0.17	−0.27	0.15	−0.32	0.08	−0.41	0.02*
Max PoP CrSO2 - Min PoP CrSO2 (%)	0.48	0.01*	0.55	0.002*	0.24	0.21	0.19	0.33

CrSO2 cerebral oxygen saturation, *NIPS* Neonatal infant pain scale, *PIPP-R* Premature Infant Pain Profile – revised, *baseline CrSO2* 10-minute average of median CrSO2 preceding the initiation of the procedure, *PoP CrSO2* 10-min average of median CrSO2 during the 10-min post-procedure, *Max PoP CrSO2* maximum CrSO2 during the 10-min post-procedure, *Min PoP CrSO2* minimum CrSO2 during the 10-min post-procedure.

*p < 0.05 is statistically significant.

## Discussion

NIRS offers the advantage of noninvasive continuous oxygen monitoring, allowing for real-time assessments and immediate intervention [18, 19]. Supportive evidence indicates that changes in CrSO2 correlate with changes in brain electrical activity in areas associated with pain perception [20, 21]. This encompasses pain induced experimentally or clinically, such as needle insertion, electrical stimulation, or dental pain, in adolescents and adults post different surgeries. Alterations in CrSO2 linked to pain perception across distinct brain regions have been reliably quantified by NIRS technology. Subsequent investigations evaluated whether the mitigation of pain responses were linked to CrSO2 fluctuations, for example, if the administration of sucrose solutions or tactile stimulation during procedures on infants was efficacious in reducing CrSO2 fluctuations [22]. These findings align with prior research utilizing other parameters to assess infant distress [14].

LPT infants are frequently managed as term infants, because of similarities in activity levels and physical characteristics, and their procedural profiles often mirror those of term infants. However, LPT infants tend to require longer hospital stays and may undergo more frequent procedural interventions than their term counterparts. Therefore, acknowledging the impact of pain responses stemming from common medical procedures in this subgroup of LTP infants is important for devising appropriate care strategies.

The LPT and term cohorts demonstrated comparable baseline CrSO2 values, establishing a benchmark for LPT infants. Previous examinations of the impact of painful procedures on CrSO2 variations in newborns, were predominantly conducted among very preterm infants and unveiled diverse trends. Some studies have reported an increase, or an initial increase followed by subsequent decrease in CrSO2, indicating an increase in cerebral blood flow in response to neuronal activity via neurovascular coupling [7, 23–26]. However, these observed patterns lacked consistency across patient groups, with an overall prevailing trend of decreased CrSO2 on average [6–8]. Our investigation revealed various patterns of changes in CrSO2, including immediate increases followed by decreases or delayed decreases across patient groups. These inconsistent patterns hinder predictability across the studied cohorts. Despite the variability, the overarching trend indicated a decrease in CrSO2. While there were changes in both increases and decreases in CrSO2, we focused primarily on the decrease aspect. This emphasis stems from the fact that lower CrSO2 values are associated with long-term neurodevelopmental outcomes [27].

In response to routine medical procedures, both LPT and term infants exhibited reduced CrSO2 values, consistent with prior findings in both very preterm and term infants [6, 7, 25]. A recent study by Kumar et al. [6] involving very-low birthweight infants noted a decline in CrSO2 estimated to range from 4% to 7%, following procedures such as heelstick or venipuncture. Remarkably, this closely aligns with the 5% reduction in CrSO2 observed in the LPT group in our study. In our investigation, 6.7% and 10% of the LPT and term infants, respectively, exhibited Min PoP CrSO2 values below the normal acceptable threshold of 60%, with no significant difference between the groups. Importantly, none of the neonates experienced CrSO2 levels below 55%, which are considered critical values. Therefore, this study affirms that routine medical procedures do not result in dangerously low CrSO2 levels.

We aimed to explore and compare these responses specifically in LPT infants, which revealed distinct reactions to painful procedures compared to term infants. These were characterized by lower variability, as indicated by a reduced IQR, a lesser decline from baseline CrSO2, and smaller fluctuations in CrSO2, as depicted by differences between the minimum and maximum CrSO2 values compared to term infants. These findings suggest a mature response to maintain cerebral oxygenation during painful stimuli in the LPT group. A previous investigation highlighted an extended latency of peak responses to painful procedures in very premature infants, attributed to slow conduction velocity and delayed synaptic response to nociceptors [25]. Enhanced neural circuit development during the late preterm period potentially influences the cortical processing of pain response [10]. We observed a similar time interval to reach the Min PoP CrSO2 between LPT and term infants. Given the comparable trend in the change of PoP CrSO2 and the time interval to reach the minimum PoP CrSO2, our findings suggest that LPT infants exhibit a response to pain processing comparable to that observed in term infants. Nevertheless, since the overall PoP CrSO2 values decreased in both groups, indicating a cortical response to common procedural pain, it is advisable to limit the number of painful procedures to the essential and avoid unnecessary repetitive interventions.

We further investigated the correlation between clinical pain scores and changes in PoP CrSO2 during painful procedures in both LPT and term infants. PIPP-R and NIPS scores are commonly employed for acute pain assessments [28]. However, a systematic review revealed that several scoring systems lack a statistically significant correlation with neurophysiological indices, such as brainwave patterns, which are currently considered direct indicators of pain perception based on brain activity [29]. A recent study in preterm infants demonstrated strong positive correlations between changes in CrSO2 and video-assessed NIPS and PIPP-R scores (*r* = 0.71 and 0.78, respectively) [6]. NIPS scores based on muscle tone changes appear to provide a clear indication of pain response. We observed an overall poor to moderate correlation between the decrease in PoP CrSO2 and higher NIPS scores, primarily in term infants, but only poor correlation in LPT infants. The lower correlation in the LPT group may stem from reduced facial expression changes in response to pain with decreasing GA [30], suggesting that facial expressions alone may not be a precise assessment method, especially in preterm infants.

Hence, there has been a shift toward incorporating physiological measures into pain assessment tools, including the PIPP-R score, which utilizes assessments of physiological, behavioral, and contextual parameters [31]. Interestingly, we did not find a substantial correlation between PIPP-R scores and changes in PoP CrSO2. This lack of correlation may be partly attributed to the contextual scores derived from GA and behavioral stage, which remain constant during the procedure and do not vary with the pain response. Therefore, LPT infants with lower PoP CrSO2 values may exhibit lower overall PIPP-R scores due to reduced responses in both physiological and behavioral aspects. The rationale as to why LPT infants demonstrated a moderate correlation between higher PIPP-R scores and a wider difference between the Max and Min PoP CrSO2, unlike term infants, without differences in group pain scores, remains unexplained. Further investigation is warranted to elucidate these intriguing findings. However, our findings indicating that NIPS and PIPP-R scores do not correlate with CrSO2, suggest that the use of pain scores alone may not accurately reflect the cortical response elicited by acute painful procedures, at least in LPT and term infants. The findings align with those of Slater et al., who observed no alteration in facial expression in 13 out of 33 assessments, with 10 of these instances (30%) exhibiting changes in CrSO2 levels [15]. Gestational and postmenstrual age dependent variations among individual components can also be observed in behavioral (posture/tone, cry, facial expression, sleep pattern) and physiological (color, respirations, heart rate, blood pressure and saturation) parameters [32].

There are several strengths of our study. It is the first to prospectively examine the impact of routine painful procedures on LPT infants. We maintained internal validity by implementing stringent controls and precise monitoring of all variables. The selection criteria were meticulously applied to include only infants with minimum confounding factors that could influence CrSO2 values, thereby ensuring an accurate portrayal of the effects of the painful procedure on CrSO2 changes. However, certain limitations should be recognized in the generalizability of our findings. First, the implemented procedures were dissimilar and characterized by their brief and minimally invasive nature, resulting in pain experiences that were neither severe nor prolonged enough to induce significant adverse effects. Second, term infants, serving as the comparative cohort, may have introduced potential bias due to abnormalities affecting cerebral autoregulation, such as variations in partial pressure of carbon dioxide (pCO2) or plasma glucose over time. Nevertheless, both groups exhibited normal baseline CrSO2 values, and no significant differences were noted between them. Furthermore, term infants often exhibited more robust crying compared to their LPT counterparts, a factor that may influence the variability of CrSO2 or contribute to elevated intrathoracic pressure, thereby leading to more pronounced fluctuations in CrSO2 compared to the LPT group. Unexpectedly, only a small number of infants (8%) in both groups received oral sucrose for non-pharmacological pain management, which cannot be justified, since the intervention is part of our routine clinical practice for comfort during medical and nursing procedures. In retrospect the lack of any comfort measure in our study that focused on pain, is a serious oversight and merits timely resolution through a continuous quality improvement initiative. However, the outcomes of our study accurately portray the direct impact of painful procedures with minimal influence from other potential confounders, particularly from analgesic medications. Last, all infants in this study were respiratory and hemodynamically stable, and may have resulted in a selective bias towards only stable infants. It is important to note that these results may manifest differently in very ill infants undergoing more intense procedures that may lead to more pronounced changes in CrSO2. However, our focus was specifically on common procedures in stable infants, which reflects common clinical practice.

In summary, both LPT and term infants demonstrated decreased CrSO2 values in response to painful procedures. Notably, LPT infants displayed milder changes in CrSO2, indicating an intact cortical response to pain comparable to term infants. The correlations between changes in CrSO2 and PIPP-R or NIPS scores were poor to moderate, highlighting the complex nature of these associations relative to gestational age.

## Supplementary information

Supplemental Table 1

Supplemental Table 2

## Data Availability

The datasets generated and/or analysed during the current study are available from the corresponding author on reasonable request.
