# Supplementary material for: A comparison of the effect of procedural pain on cerebral oxygen saturation between late preterm and term infants
Source: J Perinatol. 2024 May 24;44(11):1682–8. doi: 10.1038/s41372-024-01978-4 (PMC11519002; doi:10.1038/s41372-024-01978-4)
Supplement: Supplementary file 1 — Supplemental Table 1 [file 41372_2024_1978_MOESM1_ESM.docx]

| **Supplemental Table 1 Comparison of cerebral oxygen saturation at each minute interval during the procedure between late preterm and term infants** | | | | |
| --- | --- | --- | --- | --- |
|  | | **Late preterm (n = 30)** | **Term  (n = 30)** | ***p*** |
| CrSO2 (%) at each minute during the procedure | | | | |
|  | Minute 1 | 77.6 [75.1,81.8] | 80.0 [75.3,85.5] | 0.31 |
|  | Minute 2 | 79.5 [74.8,81.6] | 75.0 [72.1,82.1] | 0.50 |
|  | Minute 3 | 79.5 [72.9,83.5] | 79.6 [72.5,82.9] | 0.83 |
|  | Minute 4 | 80.5 [75.1,85.0] | 78.0 [73.0,85.0] | 0.56 |
|  | Minute 5 | 79.0 [75.4,84.8] | 78.5 [75.5,85.8] | 0.89 |
|  | Minute 6 | 79.8 [75.6,84.3] | 81.0 [77.0,88.6] | 0.36 |
|  | Minute 7 | 80.3 [75.4,85.6] | 82.5 [77.3,89.6] | 0.28 |
|  | Minute 8 | 79.5 [75.4,84.4] | 84.3 [77.8,88.0] | 0.34 |
|  | Minute 9 | 79.5 [76.5,85.0] | 82.5 [75.8,86.5] | 0.73 |
|  | Minute 10 | 79.0 [76.4,83.3] | 80.3 [76.3,85.5] | 0.54 |
| Abbreviation: CrSO2; cerebral oxygen saturation  Data are presented as median [25th percentile, 75th percentile].  **p* <0.05 is statistically significant. | | | | |
