# Supplementary material for: A comparison of the effect of procedural pain on cerebral oxygen saturation between late preterm and term infants
Source: J Perinatol. 2024 May 24;44(11):1682–8. doi: 10.1038/s41372-024-01978-4 (PMC11519002; doi:10.1038/s41372-024-01978-4)
Supplement: Supplementary file 2 — Supplemental Table 2 [file 41372_2024_1978_MOESM2_ESM.docx]

| **Supplemental Table 2 Comparison of the change in cerebral oxygen saturation from baseline at each minute interval during the procedure between late preterm and term infants** | | | | |
| --- | --- | --- | --- | --- |
|  | | **Late preterm (n = 30)** | **Term  (n = 30)** | ***p*** |
| Changes of CrSO2 (%) from baseline at each minute during the procedure | | | | |
|  | Minute 1 | -2.0 [-4.1,1.3] | -1.0 [-6.4,1.5] | 0.84 |
|  | Minute 2 | -1.8 [-4.5,0.6] | -3.3 [-10.1,-1.4] | 0.10 |
|  | Minute 3 | -1.0 [-3.6,1.6] | -2.6 [-7.9,0.1] | 0.24 |
|  | Minute 4 | 0.3 [-2.6,2.3] | -1.3 [-7.9,2.3] | 0.24 |
|  | Minute 5 | -0.5 [-2.0,2.6] | -0.8 [-5.1,2.3] | 0.44 |
|  | Minute 6 | 0.0 [-2.5,2.4] | 0.3 [-3.1,3.5] | 0.64 |
|  | Minute 7 | 0.8 [-2.5,2.0] | 0.3 [-1.0,2.6] | 0.90 |
|  | Minute 8 | 0.3 [-2.6,3.5] | 1.0 [-1.6,3.9] | 0.70 |
|  | Minute 9 | 0.5 [-2.5,4.0] | -0.6 [-4.0,2.5] | 0.39 |
|  | Minute 10 | 0.2 [-3.6,2.6] | -2.0 [-4.6,2.1] | 0.29 |
| Abbreviation: CrSO2; cerebral oxygen saturation  Data are presented as median [25th percentile, 75th percentile].  **p* <0.05 is statistically significant. | | | | |
